# Clinical Review of Proton Therapy in the Treatment of Unilateral Head and Neck Cancers

**DOI:** 10.14338/IJPT-D-20-00055.1

**Published:** 2021-06-25

**Authors:** Robert H. Press, Richard L. Bakst, Sonam Sharma, Rafi Kabarriti, Madhur K. Garg, Brian Yeh, Daphna Y. Gelbum, Shaakir Hasan, J. Isabelle Choi, Chris A. Barker, Arpit M. Chhabra, Charles B. Simone, Nancy Y. Lee

**Affiliations:** 1 Department of Radiation Oncology, New York Proton Center, New York, New York, USA; 2 Department of Radiation Oncology, Icahn School of Medicine at Mount Sinai, New York, New York, USA; 3 Department of Radiation Oncology, Montefiore Medical Center and Albert Einstein College of Medicine, Bronx, New York, USA; 4 Department of Radiation Oncology, Memorial Sloan Kettering Cancer Center, New York, New York, USA

**Keywords:** proton therapy, head and neck cancer, unilateral, ipsilateral, toxicities

## Abstract

Radiotherapy is a common treatment modality in the management of head and neck malignancies. In select clinical scenarios of well-lateralized tumors, radiotherapy can be delivered to the primary tumor or tumor bed and the ipsilateral nodal regions, while intentional irradiation of the contralateral neck is omitted. Proton beam therapy is an advanced radiotherapy modality that allows for the elimination of exit-dose through nontarget tissues such as the oral cavity. This dosimetric advantage is apt for unilateral treatments. By eliminating excess dose to midline and contralateral organs at risk and conforming dose around complex anatomy, proton beam therapy can reduce the risk of iatrogenic toxicities. Currently, there is no level I evidence comparing proton beam therapy to conventional photon radiation modalities for unilateral head and neck cancers. However, a growing body of retrospective and prospective evidence is now available describing the dosimetric and clinical advantages of proton beam therapy. Subsequently, the intent of this clinical review is to summarize the current evidence supporting the use of proton beam therapy in unilateral irradiation of head and neck cancers, including evaluation of disease site-specific evidence, unique challenging clinical scenarios, and ongoing clinical trials.

## Introduction

Radiation therapy is a critical component in the multidisciplinary management of head and neck malignancies. However, due to the inherent radiosensitivity of normal tissues in the head and neck region, radiation therapy for these diseases is often accompanied by significant morbidity. Common acute toxicities include mucositis, dysphagia, odynophagia, dermatitis, and xerostomia, among others. Additionally, as patients survive their disease, late complications can occur such as dental caries, osteoradionecrosis, and permanent dependence on percutaneous endoscopic gastrostomy (PEG) tubes. Many primary malignancies of the head and neck region originate in midline structures, placing lymphatic drainage on both sides of the neck at risk of subclinical disease and, therefore, necessitating the use of comprehensive bilateral neck irradiation. However, in select clinical scenarios of lateralized disease with limited risk of contralateral neck involvement, ipsilateral elective nodal irradiation is a potential option to minimize treatment morbidity while maintaining comparable locoregional control [1].

Proton beam therapy (PBT) is an advanced form of radiation therapy, and its use for treatment of head and neck cancers is rapidly growing. The inherent physical properties of protons allow for rapid dose fall-off beyond the depth of the Bragg peak. Based on these unique dosimetric capabilities, PBT is an ideal treatment modality for the irradiation of unilateral head and neck targets, providing conformal target coverage while effectively eliminating unnecessary exit dose to midline (eg, oral cavity) and contralateral normal tissues that do not require treatment for oncologic control (Figure). Minimizing exit dose has the potential to significantly reduce the acute morbidity of treatment, minimize late chronic toxicities, and better preserve patient quality of life [2]. As many patients with indications for unilateral irradiation generally carry favorable prognoses, the reduction of chronic toxicities and prioritization of long-term quality of life outcomes after treatment is of growing importance [3]. To meet this need, PBT is a promising treatment modality in this patient population. While the evidence evaluating the role of PBT in the unilateral setting is currently limited, there has been a rapid growth of data the past several years as clinical interest has increased, providing compelling support for continued investigation. In this review, we present the current level of evidence for PBT in unilateral irradiation and discuss ongoing clinical trials.

**Figure.**
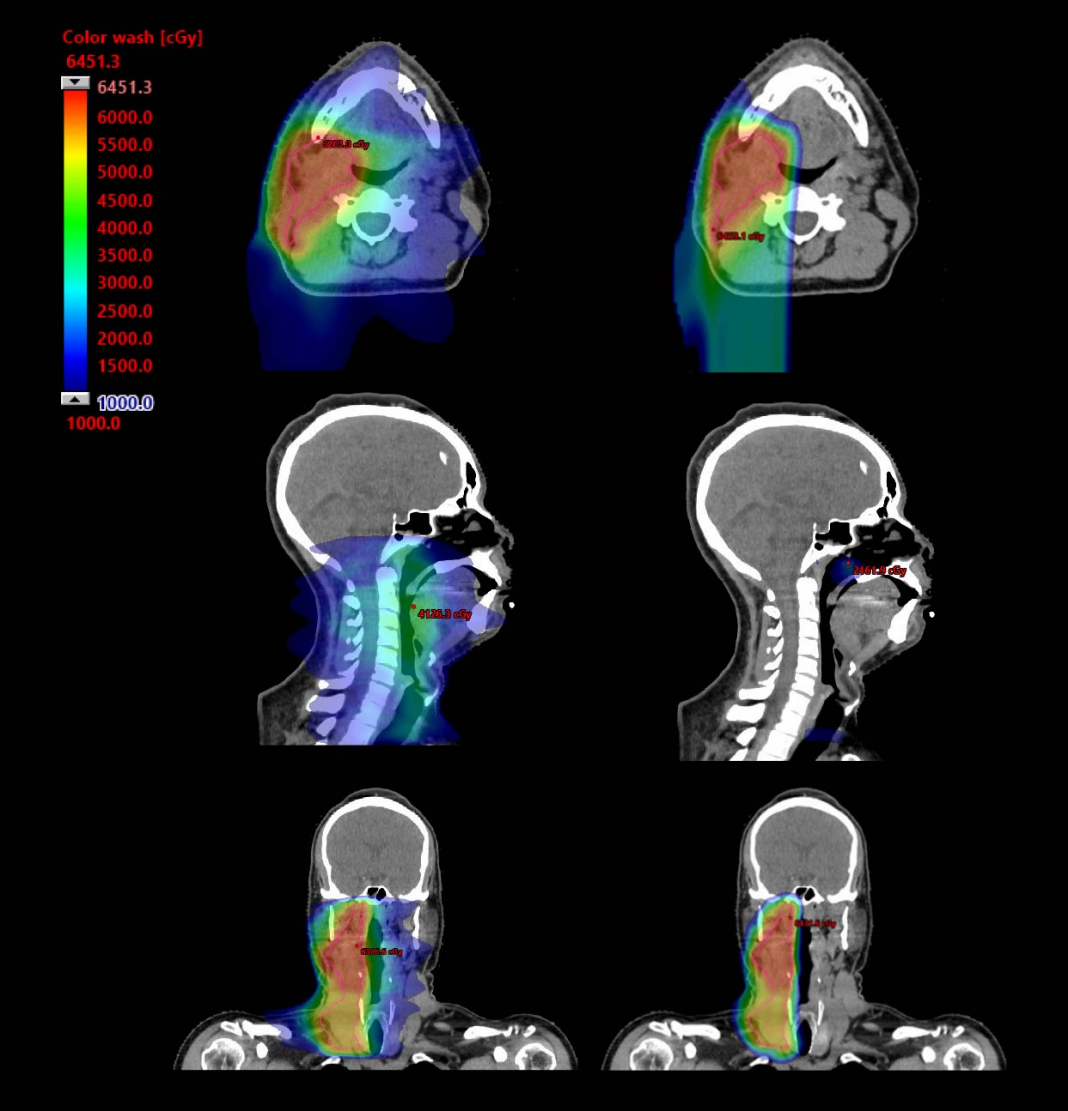
Comparison colorwash dose distribution of volumetric arc radiotherapy (VMAT) and intensity-modulated proton therapy (IMPT) for a 49-year-old man with a T2N1 HPV+ squamous cell carcinoma of the right tonsil.

## Literature Review Methods

The following literature review was conducted to identify currently published studies reporting on clinical outcomes after unilateral proton therapy in head and neck malignancies. The primary search was performed using the electronic MEDLINE PubMed database for all published literature through August 2020. Medical subject heading (MeSH) terms were used to identify peer-reviewed articles including “head and neck cancer,” “unilateral OR ipsilateral,” and “Proton therapy.” Abstracts were screened to include patients treated who had squamous cell carcinoma of the head and neck, cutaneous squamous cell carcinoma, salivary gland cancers, skull base tumors, and periocular tumors. Additional sources and clinical scenarios were subsequently identified from the references within initially captured studies. Currently active clinical trials were identified via Clinicaltrials.gov. Results were then summarized in descriptive format.

## Dosimetric Studies

Dosimetric evaluations comparing PBT and conventional photon irradiation, such as intensity-modulated radiation therapy (IMRT), are critical to appreciate the potential dosimetric advantage of protons before testing whether and the degree to which improved dosimetry translates into a clinical advantage. Several dosimetric studies have been performed that compare PBT to photon-based IMRT in the treatment of unilateral target volumes [4–7]. Kandula et al [4] reported 5 patients with ipsilateral head and neck malignancies and compared treatment plans using pencil-beam scanning (PBS) PBT and IMRT. While PBT and IMRT achieved equal target coverage and similar conformality indices, mean doses to the contralateral organs at risk (OARs) were significantly lower with PBT, including reductions in mean doses to contralateral submandibular glands (639 cGy vs 4 cGy), contralateral parotid glands (533 cGy vs 49 cGy), and oral cavity (1760 cGy vs 459 cGy), as well as decreased maximum doses to the spinal cord (3692 cGy vs 2015 cGy) and brainstem (3412 cGy vs 1388 cGy) (all *P*<0.04). They also found a significant reduction in normal tissue integral dose with PBT.

Swisher-McClure et al [5] conducted a dosimetric comparison of IMRT with PBS-PBT for 8 patients with salivary gland cancers treated to a prescription dose of 6000 cGy. In this analysis, PBS-PBT achieved lower mean doses to the ipsilateral temporal lobe (959 cGy vs 286 cGy), oral cavity (1348 cGy vs 58 cGy), contralateral parotid gland (464 cGy vs 0 cGy), ipsilateral submandibular gland (3894 cGy vs 1659 cGy), and contralateral submandibular gland (534 cGy vs 2 cGy), as well as reduction in the mandible V50 (12.8% vs 7.4%) and maximum dose to the brainstem (3090 cGy vs 710 cGy) (all *P* ≤ .01).

Detailed dosimetric analysis by Owosho et al [6] also demonstrated reduced dose to tooth-bearing regions of the mouth with PBT compared with IMRT, particularly for the contralateral molars and premolars. This difference in ipsilateral molar mean dose between the 2 modalities was most prominent for patients with parotid gland tumors (1630 cGy vs 4980 cGy) compared with oropharyngeal (3190 cGy vs 5790 cGy) or oral cavity tumors (4530 cGy vs 5960 cGy), while the difference in dose to contralateral molars was more prominent for oropharynx (22 cGy vs 2320 cGy) compared with parotid (0 cGy vs 1180 cGy) or oral cavity (4 cGy vs 1560 cGy) primary tumors. Minimizing excess radiation dose to dental structures is clinically relevant to minimize the known risk of osteoradionecrosis and mitigate late deterioration in patient quality of life [8]. Osteoradionecrosis is a predominant late complication after head and neck irradiation and in a case-matched control study has been correlated with the volume of mandible receiving a wide range of doses, most prominently 5000 cGy [9].

In summary, dosimetric comparisons demonstrate superiority of PBT compared with IMRT in minimizing excess radiation dose to contralateral OARs, particularly mean doses, while maintaining conformal target coverage. On average, PBT reduces the mean dose to the oral cavity by 75% to 95% compared to that which can be achieved with IMRT. Similarly significant, the average reductions in dose to the contralateral parotid gland is >90%, and the average reduction in dose to the contralateral submandibular gland is >99%. In certain clinical circumstances, there are also advantages to reducing dose to ipsilateral submandibular glands when intentional coverage of lymph node level Ib is not indicated, with reductions of 50% to 60% compared with IMRT [4–6] (Table 1). These advantages appear consistent across different head and neck disease sites and are most prominent for low to moderate dose levels, corresponding with the rapid distal falloff of PBT beyond prescription dose volumes. These data support further clinical investigation to determine the clinical significance of these dosimetric improvements.

**Table 1.** Summary of dosimetric studies for ipsilateral head and neck irradiation.

	**Kandula et al [4]**	**Swisher-McClure et al [5]**	**Romesser et al [7]**	**Dagan et al [10]**	**Grant et al [11]**
Radiation Modality	PBS vs IMRT	PBS vs IMRT	USPT vs IMRT	PSPT	PSPT/PBS vs EBT/IMRT
Oral cavity (mean)					
Photon	1760	1348	2060	NR	2070
Proton	458	58	94	750	460
Contralateral parotid gland (mean)					
Photon	533	464	140	NR	460
Proton	49	0	0	10	0
Contralateral submandibular gland (mean)					
Photon	639	534	410	NR	1350
Proton	4	2	0	180	0
Ipsilateral submandibular (mean)					
Photon	NR	3894	NR	NR	NR
Proton	NR	1659	NR	NR	NR
Larynx (mean)					
Photon	NR	NR	2140	NR	4430
Proton	NR	NR	1030	720	1130
Spinal cord (maximum)					
Photon	3692	NR	3630	NR	3940
Proton	2014	NR	190	NR	81
Brainstem (maximum)					
Photon	3412	3091	2970	NR	NR
Proton	1388	710	62	NR	NR

**Abbreviations:** PBS, pencil beam scanning; IMRT, intensity-modulated radiation therapy; USPT, uniform scanning proton therapy; PSPT, passive scattered proton therapy; EBT, electron beam therapy; NR, not reported.

## Clinical Outcomes

To date, there is no level I evidence supporting PBT over photon radiation modalities to treat unilateral head and neck cancers. Despite the lack of randomized data, there is a growing level of retrospectively and prospectively collected observational literature to describe clinical outcomes after unilateral PBT across a range of head and neck malignancies (Table 2).

**Table 2.** Studies evaluating proton therapy for unilateral head and neck malignancies.

**Study**	**Year**	**Type**	**Comparison With Photons**	**Disease Site**	**Patients**	**Radiation Modality**	**Dose (median)**	**Surgery (%)**	**Chemotherapy (%)**	**Follow-up (median)**	**Outcomes**	**Acute Toxicity**	**Late Toxicity**
Dagan et al [10]	2016	Prospective	No	Salivary	n = 23	PSPT	7000 cGy	70	30	NR (weekly assessments on treatment)	NR	Most patients experienced no greater than G1 toxicity. By treatment completion, G2 toxicity included mucositis (35%), dysphagia (26%), dysgeusia (23%), and xerostomia (14%). One patient with baseline G2 dysphagia developed transient G3 dysphagia. Median weight loss was 3%. No patient required PEG tube placement.	NR
Chuong et al [12]	2020	Prospective	No	Salivary	n = 105	USPT (n = 70) PBS (n = 35)	6650 cGy	70.5	20	14.3 mo	NR	Acute G2+ nausea (1.5%), dysgeusia (4.8%), xerostomia (7.6%), mucositis (10.5%), dysphagia (10.5%). Acute G3 dermatitis (10.5%), dysphagia 1.9%, fatigue 1%, and mucositis 2.9%.	NR
Holliday et al [13]	2016	Retrospective	No	Salivary (ACC)	n = 16	IMPT	6000 cGy	100	63	24.9 mo	1 patient developed LF; remaining 15 patients had no evidence of disease (LC and OS of 94%)	Cumulative acute G3 toxicity 25%, including 1 patients with G3 mucositis (6%) and 3 patients with G3 dermatitis (19%).	Cumulative late G3–4 toxicity 6.3% (1 patient developed optic neuropathy after optic nerve max dose of 7370 cGy)
Grant et al [11]	2015	Retrospective	Yes	Salivary (pediatric)	n = 24 (PBT = 13; X/E = 11)	PSPT (n = 8) IMPT (n = 5) IMRT (n = 3) IMRT/EBT (n = 7) EBT (n = 1)	6000 cGy	100	8	35 mo (PBT = 8 mo X/E = 8 y)	NR	Less G2–3 mucositis after PBT (46% vs 91%; *P* < .05), numeric reduction in G2–3 dysphagia after PBT (0% vs 27%; *P* = .08), no difference in G2–3 dermatitis (54% vs 55%; *P* = 1.0), or G2–3 otitis externa (8% vs 18%; *P* = .58).	NR
Romesser et al [7]	2016	Retrospective	Yes	Salivary (cSCC)	n = 41 (PBT = 18; IMRT = 23)	USPT (n = 18) IMRT (n = 23)	6600 cGy	90	20	8.7 mo (PBT = 4.7 mo IMRT = 16.1 mo)	1-y LC = 92.8% 1-yfreedom from distant metastases = 87.8% 1-y OS = 89.4% Nonsignificant between cohorts	Less G2–3 acute dysgeusia (5.6% vs 65.2%; *P* < .001), mucositis (16.7% vs 52.2%; *P* = .005), and nausea (11.1% vs 56.5%; *P* = .003) after PBT. Increased G2–3 dermatitis after PBT (100% vs 74%; *P* = .032)	NR
Holliday et al [27]	2016	Retrospective	No	Periorbital malignancies	n = 20	PSPT (n = 14) IMPT (n = 6)	6000 cGy	100 (orbit sparing)	55	27.1 mo	No patient developed LF, 1 patient developed regional recurrence and 1 patient developed distant metastasis	Acute G3 dermatitis (35%). No acute G3+ ocular disorders occurred.	6 patients developed late G3 ocular toxicity (30%), including 3 patients with G3 epiphora and 3 patients with G3 eyelid dysfunction/keratopathy. 5 patients developed declines in visual acuity from baseline (25%).
Manzar et al [32]	2020	Retrospective (clinical data) Prospective (PROs)	Yes	Oropharynx	n = 259 (unilateral = 44)	IMPT (n = 6) VMAT (n = 38)	7000 cGy	IMPT = 55 VMAT = 57 (entire cohort)	IMPT = 69 VMAT = 67 (entire cohort)	IMPT = 12 mo VMAT = 30 mo (entire cohort)	NR	In the unilateral cohort, there was decreased MEQ narcotic use (*P* < .05) and numeric reduction in PEG tube placement (0% vs 36.8%; *P* = .083) after IMPT. Reduced mucositis, oral pain, pharyngeal pain, weight loss, and pain after IMPT (all *P* < .05), trend toward less fatigue after IMPT (*P* = .058) but increased dermatitis and duct inflammation after IMPT (all *P* < .05). Improved PROs, including dry mouth, sticky saliva, sense of smell/taste after IMPT (all *P* < .05)	NR

**Abbreviations:** PSPT, passive scatter proton therapy; NR, not reported; G1, grade 1; G2, grade 2; G3, grade 3; PEG, percutaneous endoscopic gastrostomy; USPT, uniform scatter proton therapy; PBS, pencil beam scanning; IMPT, intensity-modulated proton therapy; ACC, adenoid cystic carcinoma; LF, local failure; LC, local control; OS, overall survival; PBT, proton beam therapy; IMRT, intensity-modulated radiation therapy; X/E, photon/electron; EBT, electron beam therapy; cSCC, cutaneous squamous cell carcinoma; PRO, patient-reported outcome; VMAT, volumetric modulated arc therapy; MEQ, morphine equivalent.

### Salivary Tumors

Salivary gland tumors consist of a heterogeneous group of histologies, including mucoepidermoid carcinoma, adenoid cystic carcinoma, adenocarcinoma, salivary duct carcinoma, and acinic cell carcinoma, among others. They can occur in major or minor salivary glands. Adjuvant radiotherapy is generally used for intermediate to high-grade, recurrent, perineural invasive, microscopic positive margins, and/or subtotally resected tumors. Major salivary gland tumors are generally positioned at shallow depths, lateral to the oral cavity and other midline structures, and effectively carry no risk of contralateral nodal spread, making these disease entities optimal targets for unilateral PBT irradiation. Dagan et al [10] prospectively collected rates of acute toxicities of patients with parotid gland tumors treated with PBT at the University of Florida. They examined 23 patients who received primary (n = 7) or adjuvant (n = 16) PBT for parotid cancers, most commonly salivary gland carcinomas. Median dose was 7000 cGy, and 7 patients received concurrent chemotherapy. The mean doses to the oral cavity (750 cGy), larynx (720 cGy), pharyngeal constrictor muscles (1950 cGy), contralateral parotid gland (10 cGy), and contralateral submandibular gland (180 cGy) were low, consistent with prior dosimetric studies. Associated rates of acute toxicities were consistently low, with most patients experiencing no greater than grade 1 mucositis, dysphagia, dysgeusia, and xerostomia. There was only 1 grade ≥3 toxicity, an event of transient grade 3 dysphagia that lasted 1 week and occurred in a patient with baseline grade 2 dysphagia. Median weight loss was 3% from baseline in the cohort, and no patients required a PEG tube. Most patients reported either no mucositis (35% to 43%) or grade 1 mucositis (17% to 30%) as per weekly evaluations during the entire treatment course. Of note, dermatitis was not described in this report.

The multi-institutional Proton Collaborative Group registry also reported prospectively collected rates of acute toxicities in patients with salivary gland tumors treated with ipsilateral PBT. In total, 105 patients (90 parotid, 15 submandibular) were treated either in the postoperative (71%) or definitive (29%) setting to a median dose of 66 Gy. The most common acute grade 2 toxicities included dermatitis (58%), esophagitis/pharyngitis (13.3%), oral mucositis (10.5%), dysphagia (8.9%), xerostomia (7.6%), dysgeusia (4.8%), and otalgia (4.8%). Grade 3 toxicities included dermatitis (10.5%), mucositis (2.9%), and dysphagia (1.9%). There were no acute grade 4 or higher toxicities [12].

Holliday et al [13] reported the MD Anderson Cancer Center (MDACC) experience using intensity-modulated proton therapy (IMPT) to treat 16 patients with unilateral adenoid cystic carcinoma. The most common subsites included the parotid gland (n = 4), lacrimal gland (n = 4), submandibular gland (n = 3), and paranasal sinus (n = 3). Median dose was 6000 cGy, and 12 patients received concurrent chemotherapy. Local control was 93.8% after a median follow-up of 2 years. Despite a cumulative acute grade 3 toxicity rate of 25%, only 1 patient (6.3%) developed acute grade 3 mucositis, with the other 3 patients experiencing acute grade 3 dermatitis (18.8%). Chronic grade 3 to 4 toxicity was only 6.3%, representing 1 patient developing optic neuropathy after receiving a maximum point dose of 7370 cGy to the affected nerve.

To date, there are only a few comparative analyses between PBT and IMRT. A 2015 study by Grant et al [11] examined 24 pediatric patients with salivary gland tumors treated with either PBT (n = 13) or conventional radiotherapy (n = 11). Once again, dosimetric superiority of PBT to many OARs was demonstrated, including reductions in doses to the oral cavity (460 cGy vs 2070 cGy) and larynx (1130 cGy vs 4430 cGy). Acute grade 2 to 3 toxicities were significantly improved with PBT, including reduced rates of mucositis (46% vs 91%), dysphagia (0% vs 27%), and weight loss (+1.2% vs –5.3%). No differences were seen in the incidence of otitis externa or dermatitis.

The largest retrospective comparison to date is the Memorial Sloan Kettering Cancer Center (MSKCC) experience reported by Romesser et al [7], which included 41 patients with major salivary gland tumors or cutaneous squamous cell carcinomas treated with PBT (n = 18) or IMRT (n = 23). The investigators shifted their practice from IMRT to PBT for ipsilateral tumors and used a matched paired analysis to compare the differences in the outcomes of these patients. Once again, PBT produced reductions in mean oral cavity dose (94cGy vs 2060 cGy), mean contralateral parotid gland dose (0 cGy vs 140 cGy), and mean contralateral submandibular gland dose (0 cGy vs 410 cGy), along with decreased median maximum doses to the brainstem (62 cGy vs 2970 cGy) and spinal cord (180 cGy vs 3630 cGy). There were no differences between cohorts with respect to locoregional control, rate of distant metastases, or overall survival. In addition, direct comparison of acute toxicities between modalities showed that PBT was associated with lower rates of grade 2 or greater acute dysgeusia (5.6% vs 65.2%), mucositis (16.7% vs 52.2%), nausea (11.1% vs 56.5%), and fatigue (5.6% vs 8.7%) (all *P* < .02). In addition, grade 3 mucositis was lower with PBT than IMRT (0% vs 8.7%), and the proportion of patients reporting no mucositis (66.7% vs 13.0%) or dysgeusia (77.8% vs 17.4%) also favored PBT (all *P* < .01). There was, of note, a greater rate of grade 2 or greater acute dermatitis with PBT than IMRT (100.0% vs 73.9%, *P* = .032), likely the effect of patients being treated with uniform scanning PBT as opposed to PBS PBT.

The current literature provides compelling single-institutional and multi-institutional level II evidence in support of PBT for salivary gland tumors with consistent minimization of acute toxicities, most prominently oral mucositis, dysphagia, and weight loss. In particular, mucositis appears dramatically reduced, with rates of acute grade ≥2 and grade ≥3 mucositis ranging from 13.4% to 46% and 0% to 6.3% after PBT [7, 10–13] compared with 52.2% to 91% and 8.7% to 28% after photon therapy, respectively [7, 11, 14, 15]. Radiation dermatitis remains the most common grade 2 to 3 toxicity related to PBT and, in some reports, is increased compared with IMRT. This finding is likely related to the lack of skin-sparing effect of protons compared with photons. This is particularly true for passive scatter PBT, an older generation of PBT with no ability to modulate the proximal dose distribution. Future studies exclusively using PBS PBT are needed to evaluate if this effect can be mitigated. Additionally, studies with longer follow-up and reports on late toxicity are lacking and require further investigation.

### Cutaneous Malignancies

Adjuvant radiotherapy for head and neck cutaneous squamous cell carcinoma is indicated for some patients with high-risk features, such as large tumor size, poorly differentiated histology, poorly defined borders, perineural invasion (PNI), lymphovascular invasion, multiple nodal involvement, extracapsular extension, patient immunosuppression, or tumor recurrence. When indicated, radiotherapy is commonly delivered to the unilateral nodal basin unless originating from a midline structure or presenting with bilateral lymph node involvement. Preauricular and intraparotid lymph nodes are common routes of regional failure for many cutaneous cancers of the head and neck. Adjuvant treatment fields, therefore, are often similar to those for salivary tumors, encompassing the parotid bed and ipsilateral cervical lymph nodes. The only study to date specifically including patients with head and neck cutaneous skin cancers treated with postoperative PBT is the previously described study by Romesser et al [7], which demonstrated significant reductions in acute dysgeusia, mucositis, nausea, and fatigue.

### Skull Base Perineural Invasion

Another promising use of PBT is in cases of perineural involvement along cranial nerves. Often, gross and/or microscopic PNI occurs along the cranial nerves ipsilateral to the primary tumor. Treatment of this disease presentation is challenging due to the need for high-dose radiotherapy around complex anatomy and in close proximity to critical neurologic OARs [16, 17]. The most commonly involved nerves include the trigeminal and facial nerves, but this varies based on the location and extent of the primary disease. Due to limited salvage options, survival is highly correlated with local control [18]. Historical studies using 3-dimensional conformal radiation therapy (3DCRT)/IMRT report local control ranging from 54% to 80% and late complication rates of 16% to 35% when treating radiographic and clinical PNI [19]. Retrospective reports using PBT to conformally cover disease extension to the base of skull have demonstrated promising local control rates of PNI upwards of 90% [20].

The major advantage of PBT in these scenarios is the ability to achieve maximal target coverage—unlike the sacrifice of tumor volume coverage that is typically necessary with photon therapy—while still meeting goal OAR constraints. PBT may therefore be more likely to achieve tumor control while minimizing the risk of late toxicities. Radiation-induced optic neuropathy is a major complication after skull base radiotherapy and can have a major impact on functional ability and quality of life. Two large studies by Kountouri et al [21] at Paul Scherrer Institute (n = 216) and Li et al [22] at Massachusetts General Hospital (n = 514) have now reported consistently low rates of radiation-induced optic neuropathy (∼1%) after high-dose PBT when the maximum dose to optic structures remains <5900 to 6000 cGy.

Additionally, lower cranial neuropathies involving cranial nerves IX, X, XI, and XII can occur after skull base irradiation. Common manifestations include chronic dysphagia, aspiration, dysphonia, and shoulder impairment. Lower cranial neuropathies are difficult to study given their low incidence (∼4% to 5%) and prolonged time to presentation (median 5 to 7 years) [23]. Despite this, they have been associated with significant detriment in long-term survivor quality of life [24] and likely remain underreported [25]. Studies with extended follow-up have correlated lower cranial neuropathies with total dose to OARs [25, 26], representing another potential opportunity to use PBS PBT's dosimetric capabilities to reduce dose to skull base foramina and pharyngeal constrictor musculature and improve long-term outcomes.

### Periorbital Tumors

Periorbital cutaneous and epithelial tumors pose a similar treatment challenge due to their location near complex critical normal tissues, including the globe, cornea, lacrimal gland, and nasolacrimal ducts. Tumors can involve the lacrimal gland, lacrimal sac, conjunctiva, and/or eyelid. Some patients may benefit from adjuvant radiotherapy for high-risk features such as positive margins/residual disease, high-grade disease, and/or positive lymph nodes. Radiotherapy can also be used for organ preservation when orbital exenteration would be otherwise required for oncologic control.

In a separate report, Holliday et al [27] described the MDACC experience treating 20 patients with periorbital tumors with PBT after globe-sparing surgery. There were no local recurrences, and only 2 patients developed regional or distant disease. Seven patients (35%) developed grade 3 radiation dermatitis, and 6 patients (30%) developed grade 3 ocular toxicities. This included 3 patients with grade 3 epiphora, 3 patients with grade 3 exposure keratopathy, and 5 patients with a decrease in visual acuity. Visual acuity declined from baseline in 2 patients from 20/20 to 20/40 as well as in 1 patient each from 20/25 to 20/80, 20/60 to 20/200, and 20/25 to 20/400. Importantly, no patients developed complete blindness, and all maintained sufficient visual acuity to perform activities of daily living. Grade 3 or higher ocular toxicity was associated with maximum dose to the ipsilateral cornea (median 4630 cGy vs 3740 cGy; *P* = .017). Notably, there was a strong numeric trend toward increased grade 3 toxicity in patients treated with passive scatter PBT (n = 14) compared with IMPT (n = 6) (43% vs 0%; *P* = .055) [27]. Dose to the conjunctiva >35 Gy and lacrimal duct system >45 Gy have been previously associated with late complications, including conjunctival telangiectasias, conjunctival keratinzation, and dry eye syndrome [28, 29]. These early reports suggest that PBT widens the therapeutic ratio in these difficult scenarios and, in select cases, provides a chance for organ preservation when conventional adjuvant radiotherapy would otherwise not be feasible.

### Oropharyngeal Cancer

Cancers of the oropharynx are another disease entity which may benefit from PBT. Well defined criteria are available to identify ideal candidates for unilateral elective nodal irradiation [30]. In addition, human papillomavirus–associated disease typically has a good prognosis, and, therefore, mitigation of late toxicities is an important consideration. As current studies are investigating methods to successfully deintensify definitive therapy, patients with well-lateralized tonsil cancer who can be appropriately treated with unilateral irradiation may potentially receive an even greater level of deintensification of toxicity with PBT. Descriptions of clinical outcomes in oropharyngeal cancer after PBT is growing; however, most comparisons to date do not distinguish between field laterality, and, thus, unilateral-specific analyses are limited.

A case-matched analysis describing the MDACC experience reported by Blanchard et al [31] examined 50 IMPT and 1000 IMRT patients, 20% of whom received unilateral irradiation. In the overall cohort, patients treated with PBT demonstrated a significantly decreased chance of requiring PEG tube placement during treatment (odds ratio [OR] = 0.53; *P* = .011) and a significant reduction in the preplanned composite endpoint of grade 3 weight loss or PEG placement at 3 months (OR = 0.44) and at 1 year (OR = 0.23; *P* < .05). There was no difference in progression free survival or overall survival.^32^

In addition, favorable patient-reported outcomes (PROs) after PBT have been demonstrated, including significant reductions in acute and subacute mucositis, xerostomia, dysgeusia, appetite, dental problems, fatigue, and physical function [32–35]. The largest study to date was a comparative analysis by Manzar et al [32] reporting the Mayo Clinic experience of provider-reported toxicities and PROs after IMPT (n = 46) and volumetric modulated arc therapy (VMAT) (n = 259). In the unilateral cohort (n = 44), significant improvements in PRO domains were identified for IMPT compared with VMAT, including dry mouth, sticky saliva, and altered taste (*P* < .05). Improvements in provider-reported toxicity favored IMPT for mucositis, oral pain, pharyngeal pain, weight loss, and fatigue, while VMAT was favored for dermatitis. There was also decreased morphine equivalent narcotic use by the end of treatment (*P* < .05) as well as a strong numeric trend in reduction of PEG-tube placement for IMPT vs VMAT (0% vs 36.8%; *P* = .083).

Bagley et al [33] described the evolution of patient-reported xerostomia scores using the Xerostomia-Related QoL Scale (XeQoLS) questionnaire in 69 patients treated with IMPT, including 12 treated with unilateral irradiation. They described expected initial worsening of scores at 6 weeks, followed by improvements over the following 2 years that approached but did not fully return to baseline. Scores were correlated with clinical factors, including mean oral cavity dose (*P* = .038), but surprisingly not laterality.

While it can be assumed that the overall magnitude of morbidity in patients treated with ipsilateral irradiation is less compared with patients treated with bilateral irradiation, the absolute difference between treatment modalities in the unilateral setting is yet to be defined. The current evidence in support of PBT, particularly benefits in PROs, is notable and warrants further investigation via randomized trials.

### Reirradiation

Locoregional recurrence remains the primary pattern of failure for head and neck cancers after definitive radiotherapy. In select patients, reirradiation provides a chance for durable tumor control but is also associated with significant high-grade toxicities—including potential grade 5 events such as carotid blowout. Accounting for the increased risk for this scenario, reirradiation treatment volumes classically target the primary/recurrent tumor or high-risk tumor bed alone without prophylactic subclinical or nodal coverage [36]. This often effectively results in unilateral target volumes. PBT is a logical treatment modality in these challenging scenarios to minimize dose overlap with previous radiotherapy courses.

There is increasing single-institutional and multi-institutional level II evidence supporting the use of PBT reirradiation. Phan et al [37] presented 60 patients prospectively enrolled and treated at MDACC with PBT for recurrent or secondary head and neck cancers. Most patients had salvage surgery (58%) and received concurrent chemotherapy (73%). The median interval from the prior course of radiotherapy was 47 months. Clinical outcomes were promising, including 1-year local control and overall survival of 68.4% and 83.8%, respectively, and grade 3 and grade 5 toxicity of 30% and 5%, respectively.

In another report, Romesser et al [38] described the multi-institutional experience of MSKCC, ProCure Proton Therapy Center, and the University of Pennsylvania. A total of 92 patients were included, 39% of whom had salvage surgery, with a median interval of 34 months. The 1-year rate of locoregional failure, with death as a competing risk, was 25.1% and the 1-year overall survival was 65.2%. Acute grade 3 or greater mucositis (9.9%), dysphagia (9.1%), esophagitis (9.1%), and dermatitis (3.3%) were low, and 2 patients developed grade 5 bleeding (2%). MSKCC is currently conducting a prospective phase II nonrandomized multiarm study for patients with recurrent and second primary head and neck cancers, including a cohort receiving full-dose PBT reirradiation. The primary endpoint of this arm is 1-year locoregional recurrence free rate.

While randomized trials to date have not demonstrated a survival benefit to chemo-reirradiation using 3DCRT [39], historical rates of high-grade toxicities approached 40% and may have undermined the improvement in locoregional control. Therefore, more conformal dose delivery with PBT may reduce late high-grade toxicities and widen the therapeutic window. Patient selection is also critical in determining which patients may benefit most from reirradiation. Dosimetric comparison studies and, ultimately, randomized trials comparing IMRT and PBT in the reirradiation setting are warranted to quantify differential outcomes and identify optimal PBT candidates.

## Randomized Trials

Ultimately, randomized trials will be important to confirm the clinical advantages of PBT in the unilateral setting. To date, there is no published level I evidence comparing PBT to IMRT in head and neck cancers. It is important to note, however, that there are no randomized trials supporting the use of IMRT to conventional 2-dimensional and 3-dimensional radiotherapy techniques in the unilateral setting, and yet IMRT is now well established as the standard of care for treating unilateral head and neck tumors.

To address the need for randomized data, MSKCC, in collaboration with ProCure Proton Therapy Center and The New York Proton Center, is currently conducting a prospective randomized phase II trial using a direct 1:1 randomization between the 2 treatment modalities (NCT02923570) [40] evaluating outcomes in patients with surgically resected salivary gland cancer, skin cancer, or melanoma requiring unilateral postoperative irradiation. The primary outcome compares the rate of acute grade 2 or greater mucositis (per the Common Terminology Criteria for Adverse Events [CTCAE], version 4.0; National Cancer Institute). The secondary outcomes assess the rate of other grade 2 or higher acute physician-reported toxicities, the rate of acute grade 2 or higher patient-reported toxicities (per the PRO-CTCAE; National Cancer Institute), the correlation between physician- and patient-reported toxicities, and the rate of late toxicities and clinical outcomes.

ARTSCAN V (NCT03829033) [41] is another ongoing multicenter randomized trial in Sweden comparing PBT to photon therapy using a 1:1 randomization. Eligibility includes T1-2, N0-1 squamous cell carcinoma of the tonsil planned for ipsilateral treatment with definitive radiotherapy alone. The primary endpoints are acute and late toxicities graded according to CTCAE.

In addition, RTOG 1008 (NCT01220583) [42] is an ongoing phase II randomized trial of postoperative radiation for high-risk salivary gland tumors with or without weekly cisplatin. While not directly studying PBT, a recent amendment allows the inclusion of patients treated with PBT. The expectation will be to assess clinical outcomes across treatment modalities, which will provide further prospective data to quantify the value of PBT in this clinical scenario.

## Discussion

Both prospectively collected single-arm studies and retrospective comparative analyses now demonstrate compelling reductions in acute toxicities in favor of PBT, particularly for mucositis, dysgeusia, xerostomia, nausea, and fatigue. In select clinical scenarios, single-arm studies demonstrate impressively low rates of chronic toxicities for optic and orbital structures and a safer path to organ preservation without compromising tumor control rates. Formal evaluations of late mucosal and glandular toxicities are still generally lacking, and the optimism for PBT to mitigate these late effects in the unilateral setting must currently be extrapolated from the limited data in the bilateral setting.

The lack of long-term follow-up is due, in part, to the contemporary nature of PBT technology along with the limited regional access resulting in fewer patients and shorter follow-up time available [43]. Late toxicities such as chronic dysphagia can be challenging to capture given the extended time to develop and/or progress symptoms as well as its association with baseline function [44]. As PBT centers become more available across the United States, ongoing prospective studies accrue, and further PBT technical advances are achieved, we expect a rapid rise in data that can more definitively evaluate the effect of PBT on reducing the risk of late toxicity.

It is important to note that acute dermatitis does not appear to be reduced by PBT and, in several studies, is reported to be worse. This may be related to the passive scatter technology used in older studies. Further research on the optimization of skin dose in PBT using PBS planning is needed. Similar to the differences in technical capabilities and resulting clinical toxicity outcomes between photon-based 3DCRT and IMRT, significant improvements in dose modulation capabilities and toxicity profiles are apparent between older generation passive scatter PBT and modern IMPT. Future studies must therefore make the distinction between different generations of technology when reporting outcomes to fully appreciate the potentials of modern PBT.

In summary, retrospective and uncontrolled prospective data support the use of PBT for patients with head and neck malignancies requiring unilateral irradiation. The extent of clinical improvement from the well-documented dosimetric advantages and comparative analyses require further quantification and confirmation in randomized trials. Several randomized studies are currently active, and we await these results to confirm the reduction in acute toxicities and quantify the possible differences in chronic toxicities.
